# Supplementary material for: An Improved Protocol for Targeted Differentiation of Primed Human Induced Pluripotent Stem Cells into HLA-G-Expressing Trophoblasts to Enable the Modeling of Placenta-Related Disorders
Source: Cells. 2023 Aug 15;12(16):2070. doi: 10.3390/cells12162070 (PMC10453333; doi:10.3390/cells12162070)
Supplement: Supplementary file 1 [file cells-12-02070-s001.zip › cells-2477087-supplementary.pdf]

Condition	BMP4 Conc. (ng/uL)	A83-01 Conc on D0 (μM)	PD173074 Conc. (μM)	A83-01 Conc. on D1 (μM)	Cells/cm^2	6 well media (mL)	BCD (cells/mL)
A	10	1	0.1	1	4000	2	19,200
E.O	10	1	0.4	7.5	3000	2.75	10,472
E.M	10	1	0.4	7.5	2500	2.75	8727

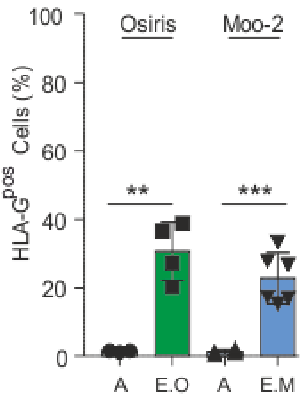

Supplemental Figure S1. BhAhP treatment leads to improved differentiation of hiPSC into HLA-G^{pos} trophoblast-like cells from different hiPSC lines. Cell seeding density optimized BhAhP treatment on two other hiPSC lines results in significantly improved HLA-G^{pos} cells. E.O – Condition E with the Osiris cell line, E.M – Condition E with the Moo-2 cell line. ** $p < 0.01$, *** $p < 0.0001$ (t-test). Data represent mean ± SD.

A

Cell line	HLA-A*		HLA-B*	
Isis	02	24	27	51
Osiris	23	30	18	44
Moo-2	01	24	08	13

C

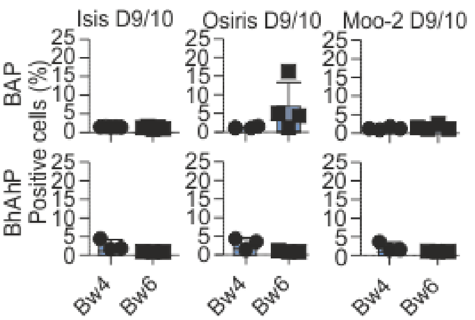

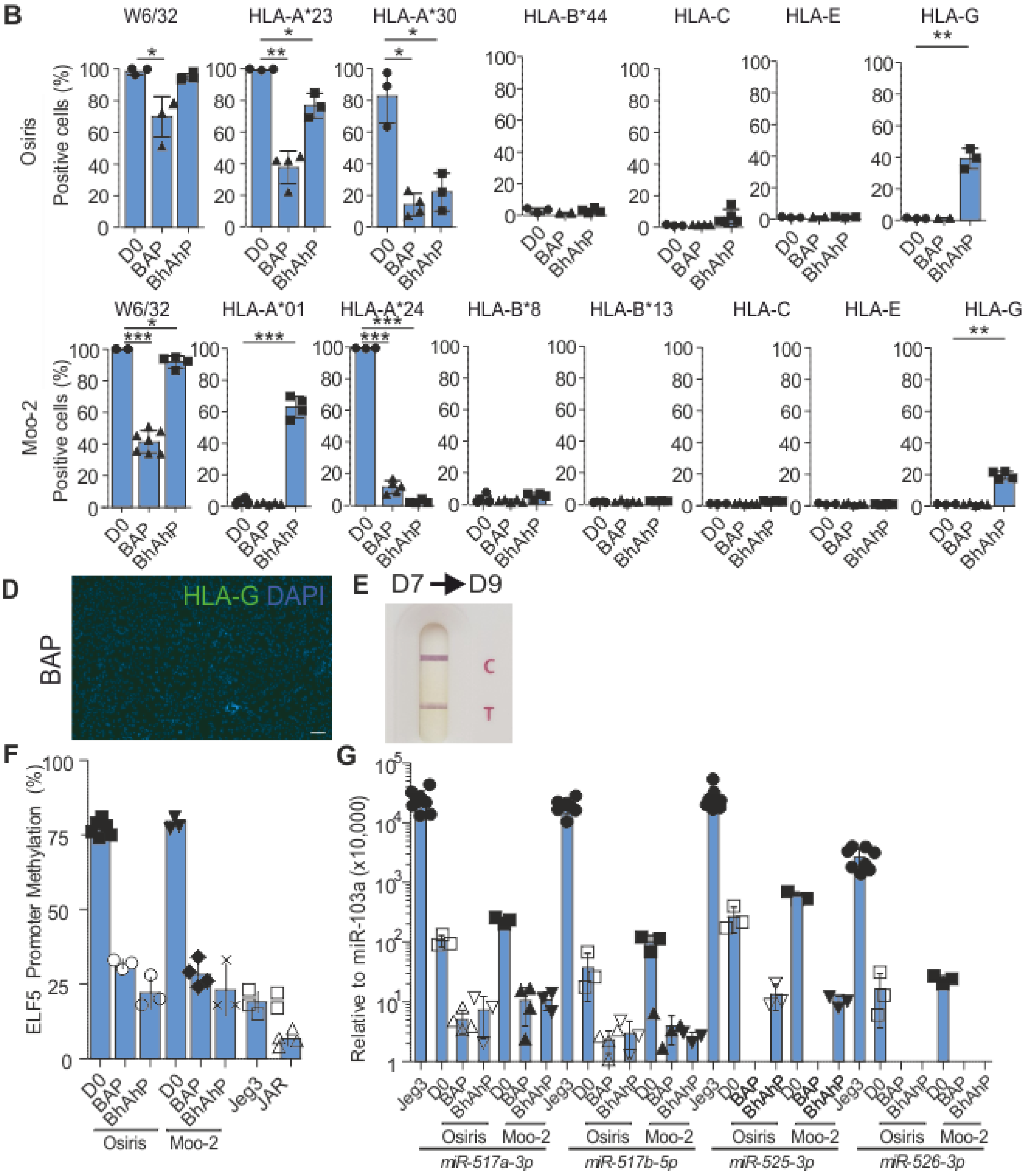

Supplemental Figure S2. HLA typing of all hiPSC and characterization data against the first trimester trophoblast criteria for the Osiris and Moo-2 cell lines. A) HLA typing of HLA-A and -B for the three hiPSC lines used in this study. B) Bar charts of flow cytometic analysis of BAP and BhAhP treated cultures with HLA-Bw4 and Bw6 antibodies. Data represent mean ± SD; ** $p < 0.01$, *** $p < 0.0001$ (t-test). C) Bar charts of flow cytometric analysis of Moo-2 and Osiris cell lines, on hiPSC (D0), BAP and BhAhP treated cells, against every HLA class I molecule. Data represent mean ± SD. D) Representative immunofluorescent image of HLA-G staining after BAP treatment. Scale bars represent 100 µm. E) Two-day conditioned medium between D7 and D9 tested positive in an hCGβ lateral flow immunoassay. F) Bar chart of average methylation across 10 CpG positions upstream of *ELF5* on hiPSC (D0), BAP and BhAhP treated hiPSC, Jeg3, and Jar cells. Data represent mean ± SD. G) Normalized expression of four miRNAs from C19MC in the hiPS cell lines Osiris and Moo-2, and primary choriocarcinoma cell line, Jeg3, over time. Results are normalized to *miR-103a* and multiplied 10,000x to ensure all logged values were positive.

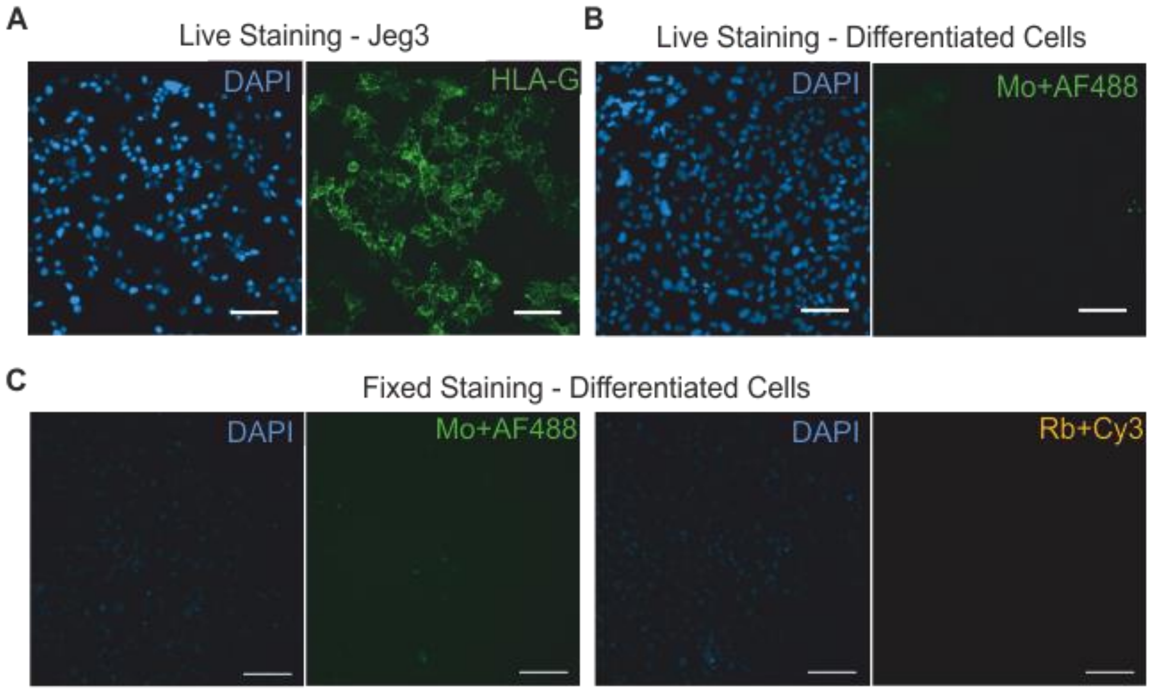

Supplemental Figure 3. Representative immunofluorescent images of Jeg3 and isotype control staining on differentiated cells. A) Immunofluorescent image after live staining Jeg3 with HLA-G. Scale bars represent 100 µm. B) Immunofluorescent image of differentiated cells after live staining with an isotype control antibody. Scale bars represent 100 µm. C) Immunofluorescent image of differentiated cells after fixation then staining with an isotype control antibody. Scale bars represent 100 µm.

Supplementary Methods and Materials

Supplemental Table S1. Flow cytometry antibody list.

Name	Conjugation	Company	Cat#	Dilution
HLA A1, A36		One Lambda	0289HA	1/55
HLA-A2 (BB7.2)		Abcam	ab74674	1/2000
HLA-A23, 24		One Lambda	0041HA	1/55
HLA A30,31		One Lambda	0273HA	1/55
HLA B8, B14		One Lambda	0332HA	1/55
HLA B13, 62, 15		One Lambda	0044HA	1/55
HLA B27, B44, 47+		One Lambda	0792AHA	1/55
HLA-C (DT-9)		Biolegend	373302	1/250
HLA-E (3D12)		Biolegend	342602	1/500
HLA-G (MEM-G/9)	APC	Exbio	1A-292-C100	1/500
HLA-G (MEM-G/9)		Exbio	11-292-M100	1/500
HLA Class I (W6/32)		Abcam	ab22432	1/500
Bw4	PE	Miltenyi	130-103-917	1/55
Bw6	PE	Miltenyi	130-099-835	1/55
Bw4	PE-Vio770	Miltenyi	130-103-920	1/55
Bw6	VioBlue	Miltenyi	130-099-846	1/55
IgG1 mouse		DAKO	X0931	
IgG2a mouse		Abcam	ab91361	
IgG2b mouse		Abcam	ab170192	
IgG mouse		Genetex	GTX35009	
Mouse IgM		DAKO	X0942	
Mouse IgG1		DAKO	X0931	
Mouse IgG2b		DAKO	X0944	
Mouse IgG2a		DAKO	X0943	
Donkey anti Mouse IgG	Alexa Fluor 488	Jackson ImmunoResearch /dianova	715-545-151	1/500
Donkey anti Mouse IgM	Alexa Fluor 488	Jackson ImmunoResearch /dianova	715-545-020	1/500
Donkey anti Mouse IgG	Alexa Fluor 647	Jackson ImmunoResearch /dianova	715-605-150	1/500

Supplemental Table S2. Immunofluorescent imaging antibody list.

Name	Conjugation	Company	Cat#	Dilution
CDX2 (CDX-88)		Abcam	AB86949	1/1
Anti human Cytokeratin 7 (CloneOV/TL-12/30)		Dako	M7018	1/150
TFAP2-C	AF647	Santa Cruz Biotechnology	sc-12762	1/150
Rabbit IgG Isotope control		Abcam	ab37415	
GATA-3 (D13C9) XP		Cell Signaling	5852	1/150
E-Cadherin (24E10)		Cell Signaling	3195S	1/750
HLA-G		Exbio	11-292-M100	1/500
Mouse IgM		DAKO	X0942	
Mouse IgG1		DAKO	X0931	
Mouse IgG2b		DAKO	X0944	
Mouse IgG2a		DAKO	X0943	
Donkey anti Mouse IgG	Alexa Fluor 488	Jackson ImmunoResearch /dianova	715-545-151	1/300
Donkey anti Rabbit IgG	Cy3	Jackson ImmunoResearch /dianova	711-165-152	1/300
Donkey anti Mouse IgG	Alexa Fluor 647	Jackson ImmunoResearch /dianova	715-605-150	1/300

Supplemental Table S3. Primer names and sequences used for bisulfite PCR sequencing, RT-qPCR, and miRNA analysis.

Primer name	Sequence
1219_hELF5-Fw	AGGAAATGATGGATATTGAATTTGAA
1221_ELF5_Fw_Seq	TTTTTTTATTATATGGGAA
1225_hELF5	GGTTATAGGTGTTTTTATTTTTATTGT
GAPDH F	CCATCTTCCAGGAGCGAGATC
GAPDH R	GCAGAGATGATGACCCTTTTGG
B-actin F	ATGTTTGAGACCTTCAACAC
B-actin R	CACGTCACACTTCATGATGG
FOXA2 F	GGGAGCGGTGAAGATGGA
FOXA2 R	TCATGTTGCTCACGGAGGAGTA
SOX17 F	CCAAGGGCGAGTCCCGTATC
SOX17 R	CACGACTTGCCCAGCATCTTG
TBXT F	ATGAGCCTCGAATCCACATAGT
TBXT R	TCCTCGTTCTGATAAGCAGTCA
miR-103a RT	GTTGGCTCTGGTGCAGGGTCCGAGGTATTCGCACCAGAGCCAACTCATAG
miR-103a F	GTAGCAGCATTGTACAGGG
miR-526b-3p RT	GTTGGCTCTGGTGCAGGGTCCGAGGTATTCGCACCAGAGCCAACGCCTCT
miR-526b-3p F	GTTTGGGAAAGTGCTTCCTTTT
miR-517a RT	GTTGGCTCTGGTGCAGGGTCCGAGGTATTCGCACCAGAGCCAACACACTC
miR-517a F	GTTTGGATCGTGCATCCTTTTA
miR-517b RT	GTTGGCTCTGGTGCAGGGTCCGAGGTATTCGCACCAGAGCCAACAGACAG
miR-517b F	GTGCCTCTAGATGGAAGCA
miR-525-3p RT	GTTGGCTCTGGTGCAGGGTCCGAGGTATTCGCACCAGAGCCAACCGCTCT
miR-525-3p F	GTTGAAGGCGCTTCCCTTT
miR universal R	GTGCAGGGTCCGAGGT
